# Progress towards HIV transmission elimination targets: model-based estimation of incidence and the extent of undiagnosed infection, Scotland, 1981 to 2022

**DOI:** 10.2807/1560-7917.ES.2025.30.36.2500164

**Published:** 2025-09-11

**Authors:** Scott A McDonald, Beth L Cullen, Lesley A Wallace, Alan Yeung, Rak Nandwani, Claudia Estcourt, Daniel Clutterbuck, Nicola Steedman, David Henderson, Kirsty Roy, Sharon J Hutchinson

**Affiliations:** 1 Glasgow Caledonian University, Glasgow, United Kingdom; 2 Public Health Scotland, Gyle Square, Edinburgh, United Kingdom; 3 Chalmers Centre, NHS Lothian, Edinburgh, United Kingdom; 4 Chief Medical Officer Directorate, Scottish Government, Edinburgh, United Kingdom

**Keywords:** HIV, Transmission, Modes of acquisition, Diagnosis, Scotland

## Abstract

**BACKGROUND:**

The global goal to end the AIDS epidemic cannot be achieved without estimates of incidence and undiagnosed infection.

**AIM:**

We aimed to estimate the timing of HIV transmission events and the number of people unaware of their diagnosis in Scotland, by mode of acquisition and migrant status.

**METHODS:**

Surveillance data from Scotland’s national HIV diagnosis database (1981–2022) linked to death and migration data was entered into the HIV Platform tool of the European Centre for Disease Prevention and Control, to back-calculate HIV incidence while imputing missing data and adjusting for reporting delay.

**RESULTS:**

We estimated 8,235 HIV transmission events between 1980 and 2022 among people living in Scotland, with an 80% reduction from 2010 to 2021 (258 to 52 events). Excluding people diagnosed outside Scotland, we estimated 4,854 (95% confidence interval (CI): 4,637–5,080) people living with HIV at the end of 2021, of whom 8.2% (396/4,854) were undiagnosed. Stratified estimates of this proportion were 6.9% for gay, bisexual and other men who have sex with men, 7.0% for people who inject drugs, 12.6% and 12.4% for heterosexuals born and not-born in the United Kingdom (UK), respectively. Including people first diagnosed with HIV outside Scotland, the overall proportion undiagnosed was 6.1% (396/6,444).

**CONCLUSION:**

Scotland is on track to meet the United Nations' diagnosis target of 95% by 2025, with the World Health Organization target of a 75% incidence reduction met since 2010. To reduce further transmission, expanded HIV testing and prevention services are necessary to better reach at-risk heterosexual individuals.

**Key public health message**

**What did you want to address in this study and why?**

The global goal to end the AIDS epidemic by 2030 cannot be achieved without a better understanding of how many people are living with HIV and of these people, how many are unaware of their diagnosis. We wanted to know how many people residing in Scotland were affected from the beginning of the epidemic to the present, and whether Scotland is on track to meet global targets.

**What have we learnt from this study?**

Using data on HIV diagnoses made from 1981 to 2022 and modelling software, we charted the time course of the HIV epidemic and estimated that about 4,850 people were living with HIV in Scotland at the end of 2021, with 6.1% unaware of their diagnosis. The estimated percentage still unaware of their diagnosis was highest among people who probably became positive through the heterosexual mode of acquisition.

**What are the implications of your findings for public health?**

Scotland is on track to meet the United Nations' target of 95% diagnosed, and has already met the WHO target of a 75% HIV incidence reduction through a national health service-funded programme involving a combination of prevention, diagnosis and treatment interventions; however, gaps and inequity in service provision need to be addressed to achieve zero people acquiring HIV in Scotland by 2030.

## Introduction

Accurate estimates of the historical number of HIV transmission events over time are invaluable for informing public health action to achieve the global target of eliminating HIV transmission by 2030 [1]. Based on such HIV incidence estimates, the number of people living with HIV (PLHIV) and the proportion of PLHIV who are unaware of their diagnosis can be derived.

At the end of 2024, an estimated 40.8 million individuals were living with HIV globally, of whom approximately 3.2 million reside in the World Health Organization (WHO) European Region [2]. According to WHO reports, 1.3 million new HIV infections occurred worldwide in 2024; this represents a 40% reduction compared with the 2010 baseline, with the target being a 75% reduction from baseline by 2025. Of these 1.3 million new HIV infections, 160,000 were among people living in the WHO European Region; this includes 17,000 in the European Union/European Economic Area (EU/EEA), reflecting a reduction of only 35% in this region since 2010 [2,3]. According to estimates, 87% of PLHIV globally are aware of their diagnosis, while in the EU/EEA countries, this figure was estimated to be 92% in 2023 [3]; thus, the EU/EEA region is regarded to be on track to meet the United Nations' diagnosis target of 95% by 2025, although so far only seven of the 30 countries (Austria, Belgium, Denmark, Finland, Iceland, Portugal and Sweden; noting four countries in this region lack data) report meeting the 95% target [3,4].

In Scotland, building on its success as one of the first countries in the world to roll out a national health service-funded HIV pre-exposure prophylaxis (PrEP) programme for those at highest risk of sexual acquisition of HIV [5], the Scottish Government has set out its ambition to eliminate HIV transmission by 2030, in addition to the WHO HIV targets to end AIDS [6,7].

A previous modelling exercise in the United Kingdom (UK) estimated that 7% of PLHIV in England (residing outside of London; note that the estimated prevalence of undiagnosed PLHIV in London is higher than in the rest of England) were unaware of their diagnosis [8]. Extrapolation of this model estimate to Scottish data produced an estimate of 500 individuals who were unaware of their diagnosis, resulting in an estimated cohort of 6,600 PLHIV in Scotland as of December 2022. As a result of limited data, however, it was not possible to estimate the proportion and number of undiagnosed individuals outside of London according to mode of acquisition. Availability of estimates derived from Scotland-specific modelling will support monitoring and – importantly – the evaluation of prevention and other initiatives that have been implemented to achieve the Scottish Government’s vision of zero people acquiring HIV within Scotland by 2030 [6]. The objective of this study was, therefore, to estimate the time series of HIV transmission events since the beginning of the epidemic in Scotland in the early 1980s using an established back-calculation modelling approach, and to estimate the proportion of PLHIV who remained undiagnosed at the end of 2021, both overall and stratified according to reported mode of acquisition and migrant status.

## Methods

### Data sources

Data on 10,474 HIV diagnoses in Scotland (i.e. individuals with a confirmed positive HIV antibody test and/or an AIDS-defining illness) were available from Scotland’s national HIV diagnosis database (maintained by Public Health Scotland (PHS)) for the period 1981 to 2022 [9], specifically: date of diagnosis (laboratory-confirmed HIV positivity), date of reporting to PHS, sex (binary variable), age, mode of acquisition (including gay, bisexual and other men who have sex with men (GBMSM), people who inject drugs (PWID), heterosexual, other and unknown), earliest recorded CD4^+^ T-cell count (normally at time of diagnosis; measured in cells/mm^3^), and country of birth (this variable was used to construct a categorical variable ‘migrant status’, a proxy for ethnicity, and the associated potential language and cultural barriers that may affect access to HIV prevention services) [10]. Data held on this database reflect voluntary testing and reporting of HIV diagnoses; while HIV infection is not a statutory notifiable disease in Scotland, completeness of reporting is very high, with laboratories that perform confirmatory HIV testing notifying PHS of any positive test results, and with quarterly checks undertaken with local laboratory/clinical teams. For the purposes of modelling transmission events, we excluded people known to have been previously diagnosed (self-reported) outside Scotland (n = 2,429). Thus, the number in the cohort for estimating incidence comprises 8,045 newly diagnosed individuals.

We defined four mode of acquisition/migrant status categories based on self-reported HIV mode of acquisition and country of birth from the national HIV diagnosis database, as follows: GBMSM, PWID, heterosexual/other mode and born in the UK, heterosexual/other mode and not born in the UK (noting there were too few HIV diagnosed persons born outside the UK in the first two categories to stratify further).

To determine vital status, date of death and possible emigration from Scotland, linkage of HIV-diagnosed persons to national administrative and death registries is routinely carried out using Community Health Index (CHI) number, a unique personal identifier for all people accessing the National Health Service in Scotland [11]. 

### Modelling approach

To estimate (i) the incidence of HIV transmission events among residents of Scotland, (ii) the total number of PLHIV and (iii) the proportion of PLHIV who are diagnosed and undiagnosed, we used the publicly available European Centre for Disease Prevention and Control (ECDC) HIV Platform tool (version 3.0.2) [12]. This tool is an interface to a validated back-calculation model and accompanying methodology for HIV incidence estimation [13-16], and has been previously deployed to estimate the proportion of PLHIV who were unaware of their diagnosis in Sweden, a country with low HIV prevalence and an epidemic initially driven by transmission through injecting drug use, similar to that of Scotland [17]. The HIV Platform tool consists of two components; the first carries out multiple imputation for missing data and adjusts for reporting delay (the time between dates of HIV diagnosis and reporting to PHS) [16]; the second component models the HIV incidence time series using a multi-state back-calculation modelling approach [15]. In this model, the time series of HIV transmission events and the time between transmission event to diagnosis are estimated, stratified by CD4^+^ T-cell count category. Through integration of data on mortality and emigration of HIV-diagnosed persons, the number of PLHIV over time is estimated. We assumed that there were no transmission events before 1980. Between 1980 and 1987, model fit was based on the total annual number of HIV diagnoses; from 1988 onwards, data on HIV diagnoses stratified by CD4^+^ T-cell count category were used for model fit.

For input to the ECDC HIV Platform tool, HIV diagnoses and HIV reporting dates were aggregated by quarter-year. We performed multiple imputation using chained equations (MICE) [18] to impute missing data on mode of acquisition (1.7% of HIV diagnosis records) and CD4^+^ T-cell count category at time of diagnosis (≥ 500, 350–499, 200–349, < 200 cells/mm^3^; 13.1% of diagnoses); we generated five imputation sets involving 20 iterations. Next, adjustment for reporting delay (which corrects for the intrinsic right-truncation of notification data that affects analysis of the most recent years) was performed on quarterly data using non-parametric reverse-time estimation [16].

Following van Sighem et al.’s approach for modelling the HIV epidemic among GBMSM in the Netherlands [15], we postulated seven time periods for the Scottish HIV epidemic; these periods are described in Supplementary Table S1. For the first two periods, diagnosis rates were estimated stratified by CD4^+^ T-cell count category and for the remaining periods, diagnosis rates were estimated aggregated over CD4^+^ T-cell category: (i) 1980 to 1983, the period in which the first AIDS cases were diagnosed; (ii) 1984 to 1987, the period in which HIV serological tests became available; (iii) 1988 to 1995, a period in which suboptimal antiretroviral therapy was available; (iv) 1996 to 1999, the period in which combination antiretroviral treatment became available; (v) 2000 to 2007; (vi) 2008 to 2019, in the latter part of this period PrEP became available (from July 2017 [9]); and (vii) 2020 to 2022, the COVID-19 pandemic period during which testing was disrupted. Diagnosis rates were modelled as a piecewise linear function of time, permitting a changing rate for the final two time periods (vi) and (vii) only. We modelled HIV incidence as a smooth function of time using cubic B-splines with four knots.

To produce estimates specific to each sex (male, female) and each mode of acquisition/migrant status category, stratified versions of the model were run, in which HIV incidence curves were separately estimated for each category, but using a distribution of reporting delay that was estimated by aggregating across all categories.

Data on emigration from Scotland, relevant for estimation of the numbers of PLHIV and diagnosed persons over time, were incorporated by simply combining with mortality (as the HIV Platform tool does not use death information for back-calculation of incidence).

Using the model-estimated HIV incidence for 2010 and 2021, we used Monte Carlo simulation to estimate the probability that Scotland had achieved the WHO target of a 75% reduction in HIV incidence by 2021, compared with the 2010 baseline. For each of 10,000 iterations the percent incidence reduction was calculated by fitting normal distributions to the point estimates and bootstrapped 95% confidence intervals (CIs) for these 2 years, and then determining the proportion of iterations in which the percent reduction was ≥ 75% (the WHO 2025 target with respect to the baseline year) and additionally for ≥ 80%.

In addition, we estimated the proportion of undiagnosed persons among *all* PLHIV in Scotland in 2021 (i.e. for the denominator of all PLHIV, we also counted persons who had been previously diagnosed elsewhere and thus acquired infection before moving to Scotland, and were known to be alive and not having emigrated by the end of 2021), by augmenting the model-estimated number of PLHIV with PLHIV who were first diagnosed elsewhere. *We expect that there are relatively few people living with untreated HIV in Scotland given the free access to modern highly effective treatment and also very few people present with AIDS.* We thus made the simplifying assumption that individuals diagnosed with HIV before moving to Scotland will either self-present or be referred to clinical services (thus prompting testing/diagnosis) relatively soon after arrival in the country. The 95% CIs around the undiagnosed proportion of all PLHIV in 2021 was approximated by dividing the 2.5th and 97.5th percentiles of the model’s estimates of the number undiagnosed by the sum of the number of PLHIV calculated by the model based on first diagnoses within Scotland and the number of first diagnoses outside Scotland. The same approach was used for mode of acquisition/migrant status specific estimates.

We used the R statistical programming environment (version 4.2.3) [19] to summarise model outputs and produce figures. We created 95% CIs within the HIV Platform tool by drawing a total of 250 bootstrap samples.

## Results

### Description of study population

Distributions over sex, mode of acquisition/migrant status category, period of HIV diagnosis, and CD4^+^ T-cell count category are shown in Table 1. The vast majority of HIV-diagnosed persons (n = 8,045) were male (5,919; 73.6%), and GBMSM mode of acquisition was the most frequently recorded (3,139, 39.0%), although the greatest proportion who had died or emigrated by the end of 2022 was among PWID (1,139; 33.7%). An appreciable proportion (2,307; 28.7%) had a very low CD4^+^ T-cell count (< 200 cells/mm^3^) at time of diagnosis; this was also the case for those who progressed to AIDS (950; 55.5%) or died/emigrated (1,137; 33.6%). The CD4^+^ T-cell count was missing for 1,057 diagnosed persons (13.1%). A total of 1,711 HIV diagnosed persons (21.3%) were known to have progressed to AIDS, (although it should be noted that AIDS reporting in Scotland is known to be incomplete from the late 1990s onwards and was suspended around 2010) and 3,384 HIV-diagnosed persons (42.1%) were known to have died (2,349; 29.2%) or otherwise emigrated (1,035; 12.9%) before or on 31 December 2022. 

**Table 1:** Description of the study population: all people diagnosed with HIV for the first time in Scotland, 1981–2022 (n = 8,045)

Subgroup	Total diagnosed		Known to have progressed to AIDS		Known to have died/emigrated before 31 Dec 2022	
	n	%	n	%	n	%
All	8,045	100.0	1,711	100.0	3,384	100.0
Males	5,919	73.6	1,331	77.8	2,511	74.2
Females	2,126	26.4	380	22.2	873	25.8
Mode of acquisition/migrant status category						
GBMSM	3,139	39.0	644	37.6	1,025	30.3
PWID	1,632	20.3	485	28.3	1,139	33.7
Heterosexual/other mode and born in UK	1,502	18.7	372	21.7	513	15.2
Heterosexual/other mode and not born in UK^a^	1,637	20.3	202	11.8	668	19.7
Unknown	135	1.7	8	0.5	39	1.2
Period of diagnosis						
1980–83	98	1.2	55	3.2	84	2.5
1984–87	1,100	13.7	489	28.6	863	25.5
1988–95	1,205	15.0	553	32.3	869	25.7
1996–99	585	7.3	143	8.4	296	8.7
2000–07	1,915	23.8	291	17.0	684	20.2
2008–19	2,838	35.3	180	10.5	578	17.1
2020–22	304	3.8	0	0	10	0.3
CD4^+^ T-cell count category (in cells/mm^3^) at time of diagnosis						
≥ 500	1,918	23.8	90	5.3	543	16.0
350–499	1,298	16.1	122	7.1	424	12.5
200–349	1,465	18.2	211	12.3	571	16.9
< 200	2,307	28.7	950	55.5	1,137	33.6
Unknown	1,057	13.1	338	19.8	709	21.0

GBMSM: gay, bisexual and other men who have sex with men; PWID: people who inject drugs; UK: United Kingdom.

**^a^** Includes 54 people with missing data on country of birth.

### Estimates of HIV incidence

**Figure 1:**
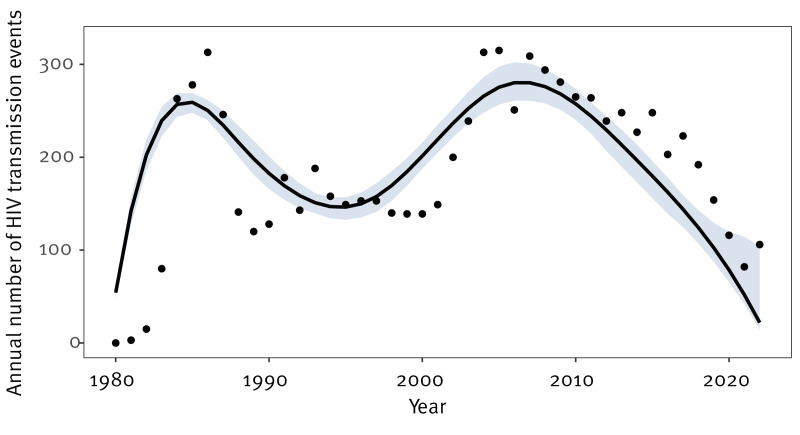
Model-estimated time series of HIV transmission events, Scotland, 1980–2022 (n = 8,235)

Over the period 1980 to 2022, the model estimated a total of 8,235 HIV transmission events (or incident infections) (Figure 1), of which 97.7% (n = 8,045) were new HIV diagnoses in Scotland (i.e. as reported to the national HIV diagnosis database). The obtained fit to the observed HIV diagnoses time series is appended for aggregated data in Supplementary Figure S1 and per mode of acquisition/migrant status category in Supplementary Figure S4, and the model-estimated time to HIV diagnosis, as function of estimated year of infection is provided in Supplementary Figure S2.

CI: confidence interval.

95% CIs are shown as shaded areas. Bootstrapped 95% CIs are shown as shading. Annual numbers of HIV diagnoses are overplotted as points.

**Figure 2:**
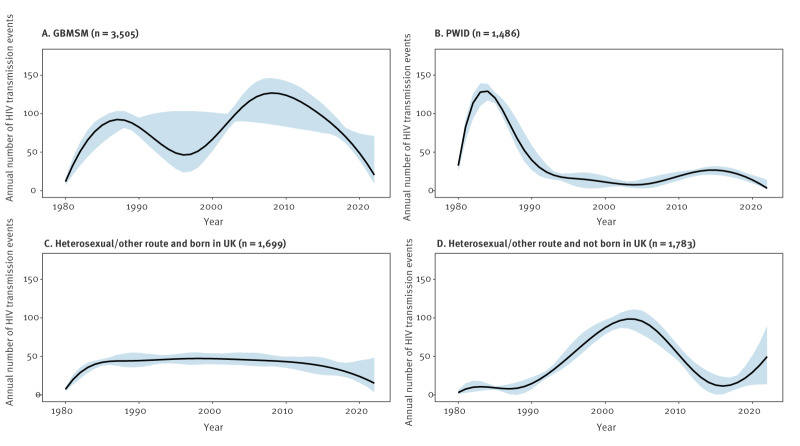
Model-estimated time series of HIV transmission events, stratified by mode of acquisition /migrant status category, Scotland, 1980–2022 (n = 8,235)

CI: confidence interval; GBMSM: gay, bisexual and other men who have sex with men; PWID: people who inject drugs; UK: United Kingdom.

Figure 1 depicts the time series of new HIV transmission events, with increasing uncertainty in estimates shown in the most recent years as an intrinsic property of the back-calculation method. Two broad peaks were apparent, of around 260 annual events in 1984 to 1985 and 280 annual events in 2006 to 2007. Modelled incidence dropped steadily after the second peak, reducing by 80% from 258 (95% CI: 240–273) transmission events in 2010 to 52 (95% CI: 42–114) in 2021. Differences were apparent between mode of acquisition/migrant status categories (Figure 2): for GBMSM, the pattern followed the overall pattern but for PWID, there was a large peak around 1983 and 1984 and smaller peak in the mid-2010s reflecting a more recent outbreak in Glasgow [20]. For the heterosexual/other mode and born in UK population subgroup, the inferred incidence time series was stable, but for the heterosexual/other mode and not born in UK subgroup, there was a broad peak between 2003 and 2005 and an indication of an upward trend in recent years following a trough in the years 2015 to 2017.

Bootstrapped 95% CIs are shown as shaded areas.

### Estimates of undiagnosed HIV infection

**Figure 3:**
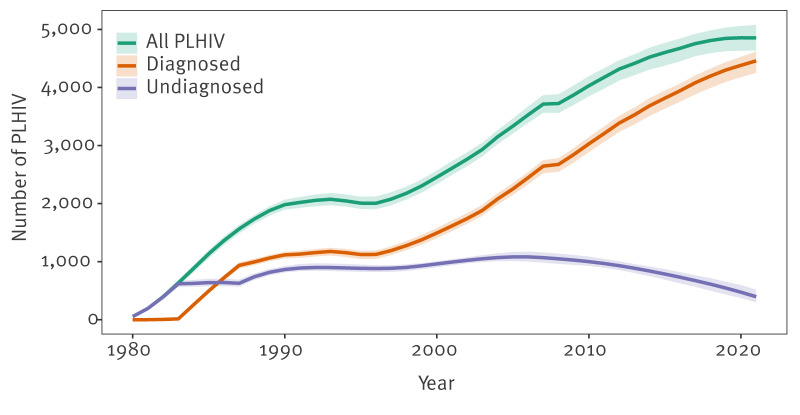
Model-estimated people living with HIV, with separate series for all, diagnosed and undiagnosed, Scotland, 1980–2021 (n = 4,854)

CI: confidence interval; PLHIV: people living with HIV.

**Figure 4:**
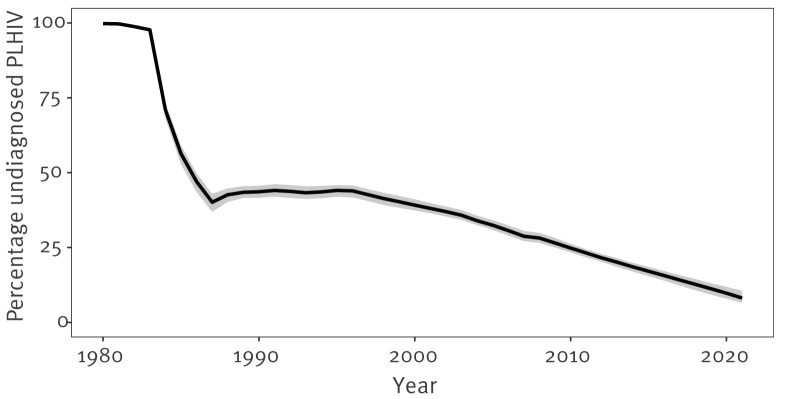
Model-estimated proportion of people living with HIV who are undiagnosed, Scotland, 1980–2021 (n = 4,854)

CI: confidence interval; PLHIV: people living with HIV.

Among the estimated number of PLHIV (excluding those who were first diagnosed elsewhere) who were residing in Scotland at the end of 2021 (n = 4,854; 95% CI: 4,637–5,080), an estimated 8.2% (n = 396; 95% CI: 303–518) remained undiagnosed at that time (Table 2, Figure 3, Figure 4). The estimated undiagnosed proportion in 2021, calculated using the same denominator but augmented with PLHIV diagnosed outside Scotland, was 6.1% (95% CI: 4.7–8.0).

**Table 2:** Model-based estimates of the number of people living with HIV in Scotland, 2001, 2011 and 2021

Subgroup	Year 2001					Year 2011					Year 2021					Year 2021		
	Estimated number of PLHIV^a^		Estimated undiagnosed			Estimated number of PLHIV^a^		Estimated undiagnosed			Estimated number of PLHIV^a^		Estimated undiagnosed			Estimated total PLHIV, including those first diagnosed outside Scotland	Estimated % undiagnosed of total PLHIV^b^	
			n	95% CI	% of PLHIV^a^			n	95% CI	% of PLHIV^a^			n	95% CI	% of PLHIV^a^			
All PLHIV	2,609	2,495–2,730	993	938–1,043	38.1	4,174	3,981–4,331	968	894–1,046	23.2	4,854	4,637–5,080	396	303–518	8.2	6,444	6.1	4.7–8.0
Mode of acquisition/migrant status category																		
GBMSM	960	818–1,314	349	268–570	36.3	1,796	1,682–1,928	344	278–392	19.1	2,344	2,203–2,493	163	123–251	6.9	3,156	5.2	3.9–8.0
PWID	466	396–526	73	50–98	15.7	344	272–427	60	41–85	17.5	354	282–424	25	14–41	7.0	407	6.1	3.4–10.1
Hetero/ other route, UK-born	666	615–724	254	222–287	38.1	965	879–1,051	213	178–252	22.1	1,154	1,054–1,288	145	102–225	12.6	1,261	11.5	8.1–17.8
Hetero/ other route, not UK-born	625	554–722	408	365–474	65.3	1,056	964–1,154	309	258–355	29.3	1,073	958–1,207	133	81–209	12.4	1,575	8.4	5.1–13.3

CI: confidence intervals; GBMSM: gay, bisexual and other men who have sex with men; PLHIV: people living with HIV; PWID: people who inject drugs; UK: United Kingdom.

^a^ Excluding those individuals living with HIV but diagnosed outside Scotland.

^b^ 95% CIs approximated by dividing the values in column ‘Estimated number of PLHIV’ by values in column ’Estimated total PLHIV, including those first diagnosed outside Scotland’

Bootstrapped 95% CIs are shown. The totals over the individual mode of acquisition/migrant status rows do not sum exactly to the ‘All PLHIV’ values because modelling was carried out stratified by population group. The 'All PLHIV' row for 2021 'Estimated total PLHIV (including those first diagnosed outside Scotland)' is larger than the sum of the values in each mode of acquisition/migrant status row, because those persons first diagnosed outside Scotland with unknown mode of acquisition/migrant status are also included.

Bootstrapped 95% CIs are shown as shaded areas.

Bootstrapped 95% CIs are shown as shading.

Model-based estimates varied by sex and mode of acquisition/migrant status category (Table 2). The percentage undiagnosed by the end of 2021 among GBMSM and PWID was estimated to be lower than for the other mode of acquisition/migrant status groups, at 6.9% (163/2,344) and 7.0% (25/354), respectively. The highest percentage undiagnosed by end 2021 was estimated for the heterosexual/other mode and born in UK category, at 12.6% (145/1,154), closely followed by the heterosexual/other mode and not born in UK group, at 12.4% (133/1,073).

### Evaluation of the World Health Organization HIV incidence target

Using Monte Carlo simulation to quantify the extent by which incidence decreased between 2010 and 2021, we estimated that a ≥ 75% reduction had a 100% estimated probability of being achieved, with a ≥ 80% reduction having a 44% estimated probability of achievement.

## Discussion

Modelled HIV incidence in Scotland decreased by at least 75% between 2010 and 2021, with 100% probability that that the WHO 2025 target had been met. An even greater reduction, of ≥ 80%, was less confident, however, with an estimated probability of 44%. Among 128 countries with available model-based incidence estimates from the Joint United Nations Programme on HIV/AIDS (UNAIDS; released in July 2021), only two had reduced new HIV transmission events by 75% between 2010 and 2020 [21]. Upon consultation of the same source 4 years later, a ≥ 75% reduction between 2010 and 2024 was indicated for five countries: Lesotho, Malawi, Nepal, Rwanda and Zimbabwe [22]. For the EU/EEA as a whole, only a 35% reduction in new HIV transmission events has been estimated between the 2010 baseline and 2023 [3].

Based on modelling, the number of undiagnosed PLHIV in Scotland (excluding individuals diagnosed outside the country) in 2021 was estimated to be low, at 396 (8.2% of PLHIV). Almost half of this number were GBMSM; the next largest category were heterosexual people born in the UK. When integrating into the denominator those PLHIV who were diagnosed outside Scotland and thus excluded from the modelling (thus providing a closer estimate of the true proportion of all PLHIV), the estimated proportion undiagnosed dropped further, to 6.1% (95% CI: 4.7–8.0).

The inferred incidence time series aggregating over mode of acquisition/migrant status indicated two peaks: around 1984 to 1985 and 2006 to 2007. The major reduction following the first peak coincided with (i) community-led awareness-raising later backed by national media campaigns of the 1980s, (ii) the introduction of azidothymidine as HIV treatment in 1987, and (iii) the advent of HIV combination therapies from the mid-1990s [23-25]. The decline following the second peak suggests an impact on HIV acquisition due to two driving factors: the first is a change to HIV treatment guidelines (i.e. antiretroviral therapy (ART) made available at all HIV stages independently of CD4^+^ T-cell cell count) [26,27], which was based on trials showing a benefit of early ART [28,29]. The second factor is the implementation of an NHS-funded HIV PrEP programme in Scotland in July 2017 [30], most clearly visible for the GBMSM mode of acquisition.

We calculated that 6,444 PLHIV were living in Scotland by the end of 2021, including those previously diagnosed outside of Scotland [8]. These estimates are comparable to those produced by a previous UK-wide independent modelling exercise, which proposed 6,613 PLHIV in Scotland at the end of 2022, of whom 7% (n = 463) had not yet been diagnosed [9]. Unlinked anonymous testing of residual specimens is planned to further corroborate these estimates in Scotland.

The current study has demonstrated how model-based estimates of incidence and undiagnosed proportion can be derived using population-wide data from HIV/AIDS surveillance systems, which necessarily rely on diagnosis reporting [3]. Furthermore, stratified model-based incidence estimates can complement epidemiological studies aimed at specific modes of acquisition and contribute to understanding the ensuing results, such as a prospective cohort study conducted among England’s GBMSM [31] and a recent modelling study of the effect of prevention initiatives among GBMSM in the UK [32].

As with all modelling approaches, our estimates depend on several assumptions and the quality and completeness of the input data. However, Scotland is among a relatively small number of countries with high-quality national-level surveillance data extending back to the beginning of the HIV epidemic [8,17]. We addressed completeness through standard imputation methods to handle missing data on mode of acquisition/migrant status and CD4^+^ T-cell count, and additionally adjusted for reporting delay. The back-calculation model necessarily assumes that all transmission events occurred among residents in Scotland. For those individuals who migrated to and were first diagnosed in Scotland, the possibility of the transmission event occurring before their move to Scotland cannot be excluded; however, this would probably have only a small effect on the incidence curves. People diagnosed with HIV before moving to Scotland were not counted directly in the modelling of the diagnosis time series as we did not have information on their date of first diagnosis (outside Scotland) but also because they acquired their infection outside Scotland. There is no impact from this omission on our outcomes as we are not modelling transmission per se but instead back-calculating incidence from the diagnosis time series; thus, the model estimated incidence time series reflects transmission stemming from all those living with HIV in Scotland (including those diagnosed first outside the country). Estimated incidence in the short period of time before the COVID-19 pandemic in Scotland might have been affected if the number of individuals diagnosed with HIV between 2020 and 2022 was smaller, because of reducing testing, than if the pandemic had not occurred. However, we believe that the pandemic influence was minimal, as the downward trend observed since 2017 in the number of newly diagnosed individuals continued into the pandemic period, and because diagnosis rates were modelled as a piecewise function of time, with the final period set to the years 2020 to 2022. Finally, because the model used only data on mortality/emigration of diagnosed individuals, annual HIV incidence may be slightly under-estimated due to migration or death of undiagnosed persons.

## Conclusions

Scotland is one of few countries to evidence major progress on HIV transmission elimination, with the WHO 75% reduction in incidence target met and the 95% diagnosis target close to being achieved. To reduce the incidence of further transmission events, heterosexual people at high risk of HIV acquisition should be a focus for case finding, testing, diagnosis and contact tracing, combined with prevention interventions for HIV-negative sex partners such as HIV PrEP. Opt-out testing in the emergency department setting – which has been recently piloted in Scotland – may also improve rates of diagnosis. As such, these findings will have implications for the development, implementation and evaluation of prevention and testing initiatives which form a central part of the Scottish Government HIV Transmission Elimination Delivery Plan.

## Data Availability

The surveillance data can be sourced from Public Health Scotland, in line with their policy on statistical disclosure (https://publichealthscotland.scot/publications/public-health-scotland-statistical-disclosure-protocol/public-health-scotland-statistical-disclosure-protocol-version-22).

## Supplementary Data

462000 Supplement
