# Supplementary material for: Progress towards HIV transmission elimination targets: model-based estimation of incidence and the extent of undiagnosed infection, Scotland, 1981 to 2022
Source: Euro Surveill. 2025 Sep 11;30(36):2500164. doi: 10.2807/1560-7917.ES.2025.30.36.2500164 (PMC12432492; doi:10.2807/1560-7917.ES.2025.30.36.2500164)
Supplement: Supplement [file 25-00164_HUTCHINSON_Supplement.pdf]

SUPPLEMENTARY MATERIAL

This supplementary material is hosted by Eurosurveillance as supporting information alongside the article *Progress towards HIV transmission elimination targets: model-based estimation of incidence and the extent of undiagnosed infection, Scotland, 1981 to 2022*, on behalf of the authors, who remain responsible for the accuracy and appropriateness of the content. The same standards for ethics, copyright, attributions and permissions as for the article apply. Supplements are not edited by Eurosurveillance and the journal is not responsible for the maintenance of any links or email addresses provided therein.

Table S1. Specification of calendar period-dependent HIV diagnosis rates in the ECDC HIV Platform tool.

Period	Allow rate in period to start from a new baseline value?	Allow rates in period to differ by CD4 count category?	Allow rates to change during period?
**1980-1983**	No	Yes	No
**1984-1987**	Yes	Yes	No
**1988-1995**	Yes	No	No
**1996-1999**	No	No	No
**2000-2007**	No	No	Yes
**2008-2019**	No	No	Yes
**2020-2022**	No	No	No

Figure S1. Model fit to observed HIV diagnoses, Scotland 1981-2022. Bootstrapped 95% CIs are shown as shaded area.

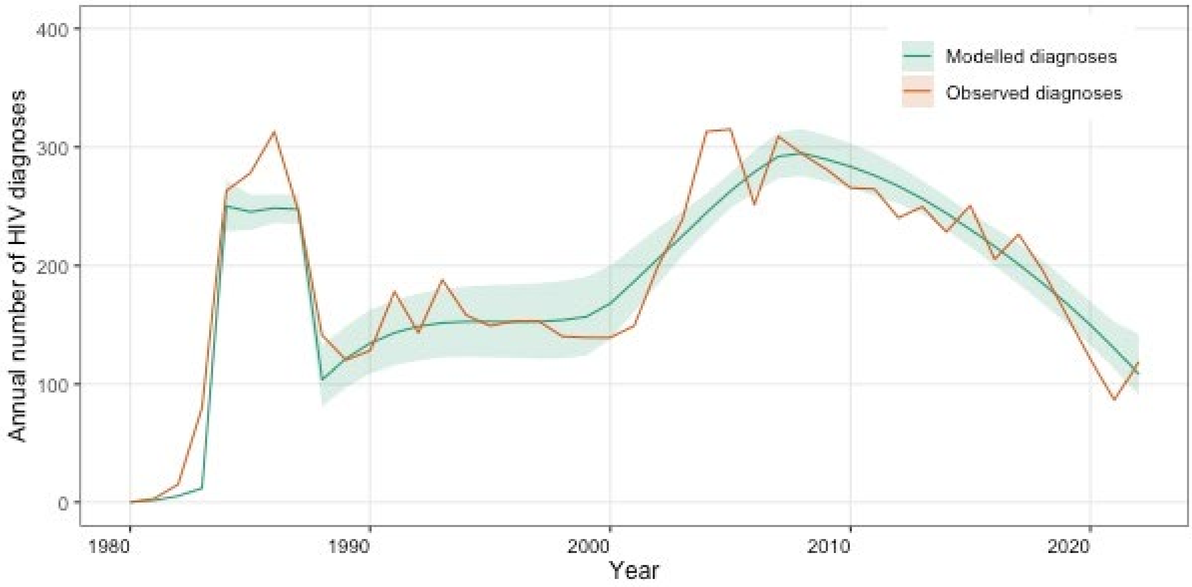

Figure S2. Model-estimated time to diagnosis as function of year of HIV infection, Scotland 1980-2022**.** Bootstrapped 95% CIs are shown as shaded area.

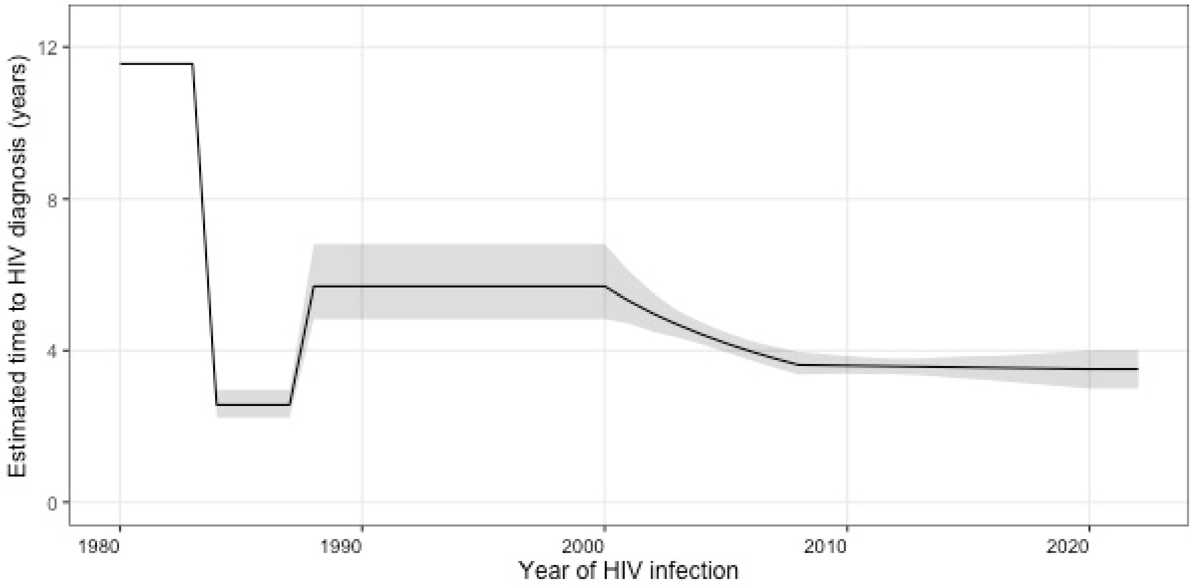

Figure S3. Model-estimated number of people living with HIV (PLHIV), with separate series for all PLHIV, diagnosed PLHIV and undiagnosed PHLIV, Scotland 1980-2021, stratified by mode of acquisition/migrant status category: GBMSM (panel A); PWID (panel B); heterosexual/other mode & born in UK (panel C); heterosexual/other mode & not born in UK (panel D). Boot-strapped 95% CIs are shown as shaded areas.

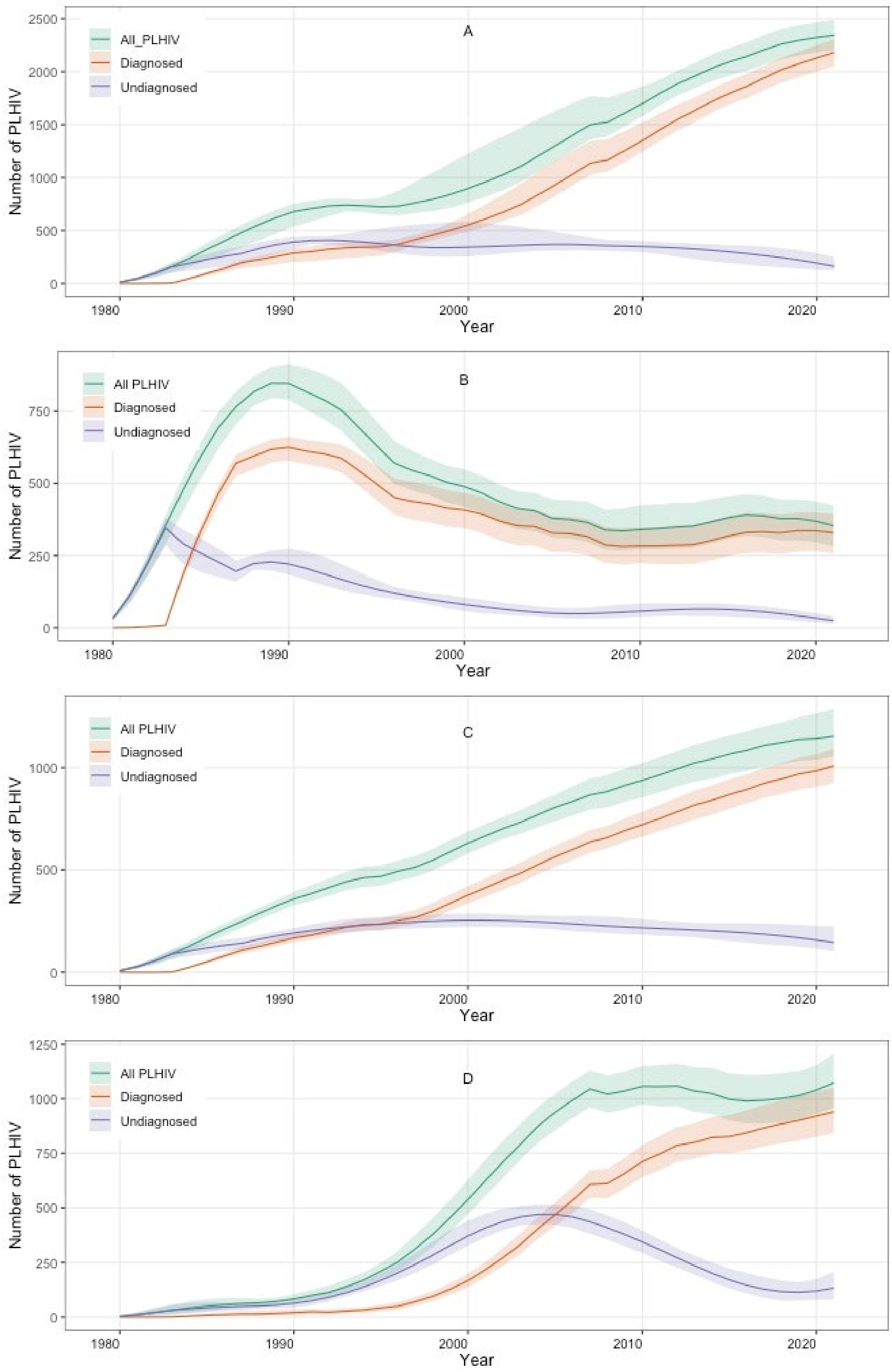

Figure S4. Model fit to observed HIV diagnoses, Scotland 1981-2022 stratified by mode of acquisition/migrant status category: GBMSM (panel A); PWID (panel B); heterosexual/other mode & born in UK (panel C); heterosexual/other mode & not born in UK (panel D). Bootstrapped 95% CIs are shown as shaded areas.

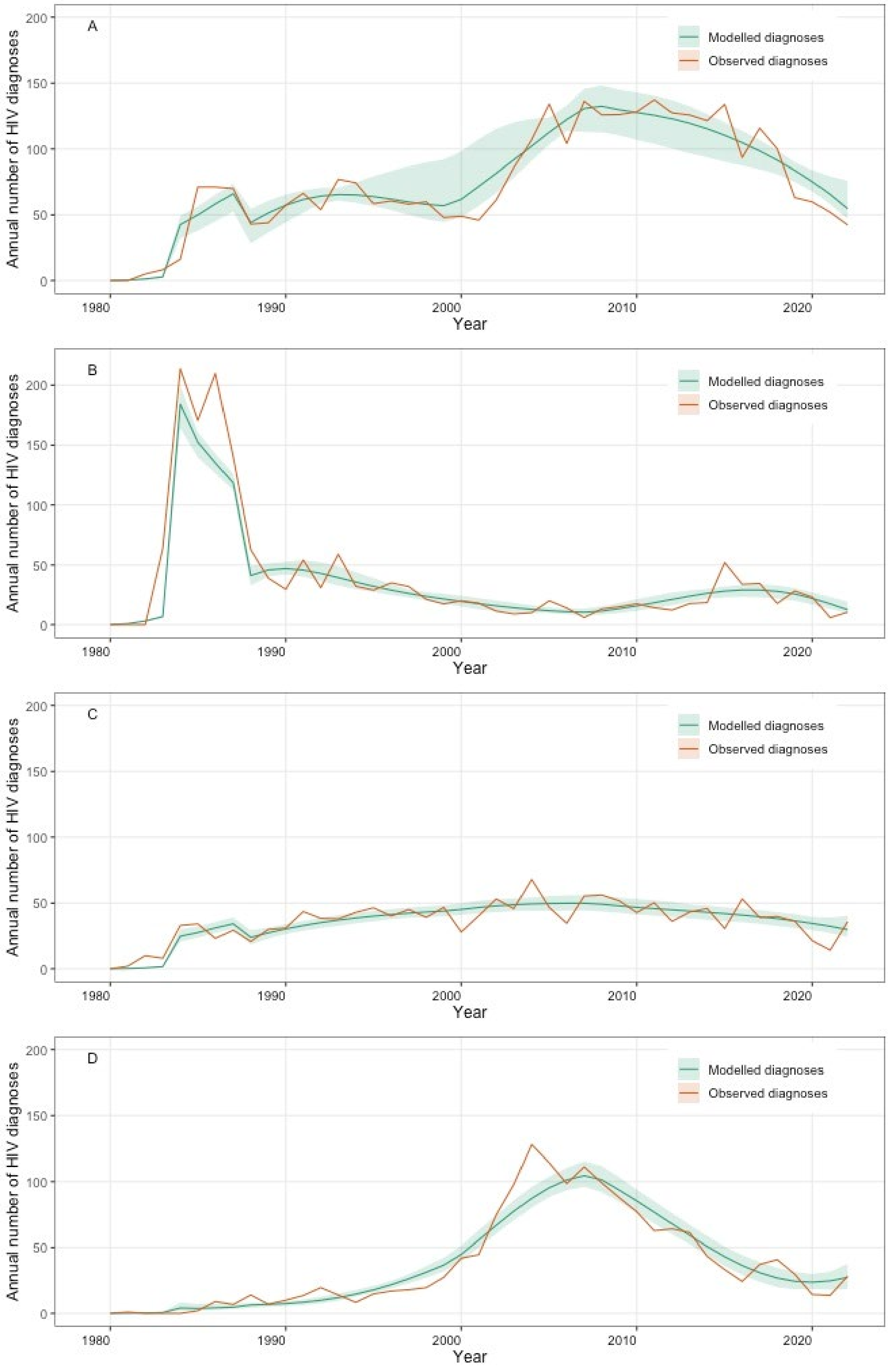

Classified as ECDC NORMAL
